# Prognostic analysis and outcomes of metastatic pancreatic cancer patients receiving *nab*‐paclitaxel plus gemcitabine as second or later‐line treatment

**DOI:** 10.1002/cam4.7345

**Published:** 2024-06-25

**Authors:** Guido Giordano, Michele Milella, Matteo Landriscina, Francesca Bergamo, Giuseppe Tirino, Antonio Santaniello, Alberto Zaniboni, Enrico Vasile, Ferdinando De Vita, Giovanni Lo Re, Vanja Vaccaro, Elisa Giommoni, Donato Natale, Raffaele Conca, Daniele Santini, Luigi Maiorino, Gianni Sanna, Vincenzo Ricci, Aldo Iop, Vincenzo Montesarchio, Letizia Procaccio, Silvia Noventa, Roberto Bianco, Antonio Febbraro, Sara Lonardi, Giampaolo Tortora, Isabella Sperduti, Davide Melisi

**Affiliations:** ^1^ Unit of Medical Oncology and Biomolecular Therapy, Department of Medical and Surgical Sciences University of Foggia Foggia Italy; ^2^ Section of Oncology, Department of Medicine University of Verona School of Medicine and Verona University Hospital Trust Verona Italy; ^3^ Department of Oncology Veneto Institute of Oncology IRCCS Padova Italy; ^4^ Unit of Medical Oncology, Sacro Cuore di Gesu'—Fatebenefratelli Hospital Benevento Italy; ^5^ Department of Clinical Medicine and Surgery University of Naples "Federico II" Naples Italy; ^6^ Medical Oncology Unit Fondazione Poliambulanza Brescia Italy; ^7^ Unit of Medical Oncology 2 Azienda Ospedaliero‐Universitaria Pisana Pisa Italy; ^8^ Division of Medical Oncology, Department of Precision Medicine University of Campania "L. Vanvitelli" Naples Italy; ^9^ Medical Oncology and Immune‐Related Tumors Centro di Riferimento Oncologico di Aviano (CRO), IRCCS Aviano Italy; ^10^ Medical Oncology 1 IRCCS Regina Elena National Cancer Institute Rome Italy; ^11^ Medical Oncology Unit Careggi University Hospital Florence Italy; ^12^ Ospedale San Massimo Penne Italy; ^13^ Division of Medical Oncology, Department of Onco‐Hematology IRCCS‐CROB, Referral Cancer Center of Basilicata Rionero in Vulture Italy; ^14^ Medical Oncology A University of Rome, Policlinico Umberto I, "La Sapienza Rome Italy; ^15^ Medical Oncology Unit San Gennaro Hospital Naples Italy; ^16^ Medical Oncology Istituto Ospedaliero dell'Università di Sassari Sassari Italy; ^17^ Medical Oncology Unit Azienda Ospedaliera di Rilievo Nazionale ‘San Pio’ Benevento Italy; ^18^ Department of Oncology Azienda Sanitaria Universitaria Giuliano Isontina (ASUGI) Trieste Italy; ^19^ Oncology Unit—A.O.R.N. dei Colli Monaldi Hospital Naples Italy; ^20^ Oncologia Medica Fondazione Policlinico Universitario Gemelli IRCCS Rome Italy; ^21^ Oncologia Medica Università Cattolica del Sacro Cuore Rome Italy; ^22^ Biostatistical Unit IRCCS Regina Elena National Cancer Institute, Istituti Fisioterapici Ospitalieri Rome Italy; ^23^ Investigational Cancer Therapeutics Clinical Unit Azienda Ospedaliera Universitaria Integrata Verona Italy; ^24^ Digestive Molecular Clinical Oncology Research Unit University of Verona Verona Italy

**Keywords:** FOLFIRINOX, gemcitabine, *nab*‐paclitaxel, pancreatic cancer, prognostic model, second‐line chemotherapy

## Abstract

**Background:**

Pancreatic cancer (PC) first‐line therapy often consists of polychemotherapy regimens, but choosing a second‐line therapy after disease progression, especially following first‐line FOLFIRINOX, remains a clinical challenge. This study presents results from a large, multicenter, retrospective analysis of Italian patients with metastatic PC (mPC) treated with Nab‐paclitaxel/Gemcitabine (AG) as second or later line of treatment. Main objective of the study is to identify prognostic factors that could inform treatment decisions.

**Methods:**

The study included 160 mPC patients treated with AG in 17 Italian institutions. AG was administered according to labelling dose, until disease progression, unacceptable toxicity or patient refusal. Variations in schedules, dose modifications, supportive measures, and response evaluation were determined by individual clinicians' practice.

**Results:**

AG was well‐tolerated and exhibited promising clinical activity. The overall response rate (ORR) and the disease control rate (DCR) were 22.5% and 45.6%, respectively. Median progression‐free survival (PFS) and overall survival (OS) were 3.9 and 6.8 months, respectively. Among the patients who received AG as a second‐line therapy (*n* = 111, 66.9%), median PFS and OS were 4.2 and 7.4 months, respectively. Notably, in the 76 patients (68%) receiving AG after first‐line FOLFIRINOX, an ORR of 19.7% and a DCR of 46.0% were observed, resulting in a median PFS of 3.5 and median OS of 5.7 months. The study identified specific clinical or laboratory parameters (LDH, NLR, fasting serum glucose, liver metastases, ECOG PS, and first‐line PFS) as independent prognostic factors at multivariate level. These factors were used to create a prognostic nomogram that divided patients into three risk classes, helping to predict second‐line OS and PFS.

**Conclusions:**

This study represents the largest real‐world population of mPC patients treated with AG as a second or later line of therapy. It supports the feasibility of this regimen following first‐line FOLFIRINOX, particularly in patients with specific clinical and laboratory characteristics who derived prolonged benefit from first‐line therapy.

## 1 INTRODUCTION

Pancreatic cancer (PC) is a lethal disease with an extremely poor prognosis confirmed by the 5‐year survival rate of approximately 10%.^1^, ^2^ The majority of patients are diagnosed with unresectable, locally advanced, or metastatic disease, making palliative chemotherapy the preferred treatment option in this context.^3^ Over the past decade, metastatic pancreatic cancer (mPC) treatment landscape has significantly evolved, primarily due to the implementation of more potent first‐line polychemotherapy regimens in clinical practice.

FOLFIRINOX and *nab*‐paclitaxel/gemcitabine (AG) have shown to significantly prolong survival, as compared to Gemcitabine alone in phase III randomized trials.^4^, ^5^ Moreover, a four‐drug combination of cisplatin, *nab*‐paclitaxel, capecitabine and gemcitabine (PAXG) has also shown better survival and response rate versus AG in a randomized phase II study.^6^ The enduring discussion in the scientific community regarding the optimal choice between triple and double chemotherapeutic agent combinations for mPC treatment has been recently elucidated by the results of the NAPOLI‐3 phase 3, randomized, controlled trial.

The study showed that the combination of liposomal irinotecan prolonged median overall survival (OS) and progression‐free survival (PFS) compared to AG, while maintaining a manageable safety profile. These encouraging outcomes suggest that NALIRIFOX could potentially become a new standard of care for the initial treatment of mPC.^7^

Among patients who progress after first‐line chemotherapy, approximately 50% receive second‐line systemic treatment.^8^, ^9^ One randomized trial and several meta‐analyses conducted in the gemcitabine monotherapy era have suggested that second‐line chemotherapy may impact on survival (vs. BSC) and that multiagent, fluoropyrimidine‐based, regimens may obtain better results.^10^, ^11^, ^12^ In the current scenario, in which AG and FOLFIRINOX/NALIRIFOX are the preferred first‐line regimens,^13^ national and international guidelines recommend differential second‐line treatment paths, depending on the first‐line regimen received.^14^, ^15^, ^16^ Fluoropyrimidines, alone or in combination represent the preferred second‐line therapeutic choices in patients progressed to first‐line AG;^17^, ^18^ among fluoropyrimidine‐based doublets, the OFF regimen and *nal*‐IRI and 5‐FU/folinic acid (nal‐IRI/FUFA) are supported by randomized phase III trials.^19^, ^20^ However, a second phase III trial (PANCREOX) employing a different regimen (mFOLFOX6) has not shown an advantage for the addition of oxaliplatin, while recent meta‐analyses (also including the NAPOLI‐1 trial) have suggested a potential advantage for irinotecan‐containing regimens.^21^, ^22^, ^23^, ^24^ In patients who have been exposed to FOLFIRINOX in first line, gemcitabine monotherapy and AG are supported by retrospective evidence or single‐arm phase II studies^25^, ^26^, ^27^, ^28^, ^29^, ^30^, ^31^, ^32^, ^33^; although no formal, randomized, comparison between gemcitabine monotherapy and AG exists in the post‐FOLFIRINOX second‐line setting, retrospective analyses suggest a potential advantage for the AG combination.^34^

Regardless of the regimen employed, a thorough analysis of prognostic/predictive factors that could assist in tailoring second‐line treatment to the subgroup of patients likely to benefit the most is currently lacking. In this context, we report the results of a large, multicentre, retrospective analysis conducted in Italy including mPC patients treated with AG as their second or further line in a real‐world setting with a focus on clinical outcomes and prognostic factors.

## 2 PATIENTS AND METHODS

Patients with mPC treated with AG combination as second or further line of therapy at 17 Italian Institutions between September 2011 and January 2015 were retrospectively identified and included in this analysis. Inclusion criteria were: cytologically or histologically confirmed metastatic pancreatic cancer; age ≥ 18 years; administration of at least one cycle of AG as second or later line of treatment; availability of clinic‐pathological and laboratory parameters at baseline of AG treatment; availability of response evaluation and survival data. Treatment was performed until disease progression, unacceptable toxicity or patient refusal. Starting schedule variations, dose modifications, supportive measures (i.e., use of Granulocyte Colony Stimulating Factor, G‐CSF or Erythropoietin, EPO) were allowed and applied according to the single clinicians' routinely practice, as well as the timing and the modality of response evaluation. All patients included have provided written informed consent to use clinical data for scientific purposes before treatment initiation and this allowed clinicians to administer AG as second or further line of therapy, perform laboratory analysis and collect data in a specific database. The Coordinating Site's institutional board (CE Lazio 1, Prot. 1439/2014) approved the study.

### 2.1 Statistical analysis

Data were censored on July 31st 2015 and primary endpoint of this analysis was Overall Survival (OS), defined as the time elapsed between the Day 1 of the first cycle of AG treatment and death or last follow up visit. Secondary endpoints were Progression Free Survival (PFS, measured from the time of Day 1 of the first cycle of AG treatment and disease progression or death, whichever occurred first) and Disease Control Rate defined as Complete Response + Partial Response + Stable Disease (evaluated by Response Evaluation Criteria In Solid Tumors, RECIST v 1.0).^35^ In the PFS evaluation, patients in which oxaliplatin and/or irinotecan was omitted during FOLFIRINOX (classic or modified schedule), were considered still on first‐line treatment until disease progression occurred. Survival curves were estimated using the Kaplan–Meier product‐limit method and the Log‐Rank test was performed to compare survival among different groups of patients.

Univariate and multivariate Cox proportional hazard model was developed using the stepwise regression (forward selection, enter limit and remove limit, *p* = 0.10 and *p* = 0.15, respectively), to identify independent predictors of outcomes. All the continuous variables were dichotomized according to prognosis with the maximally selected log‐rank statistics analysis (the best “splitter” cut‐off is determined). Factors included in univariate/multivariate analysis both for OS and PFS were: age (using a threshold of 70 years to define elderly patients), sex, primary tumor location (body‐tail vs. head), location of metastatic sites (other metastatic sites vs. liver metastases vs. liver plus other sites), number of metastatic sites (1 vs. ≥2), prior radical surgery, Eastern Cooperative Oncology Group Performance Status (ECOG PS 0–1 vs. 2), first line therapy (FOLFIRINOX vs. Gemcitabine based), response to first line therapy (Response/Stable vs. Progression), progression free survival during first line therapy (< 11 months vs. ≥11 months), biliary stent implant (no vs. yes), baseline CA19.9 (< 2034 U/I vs. ≥2034 U/I), baseline bilirubin (<0.82 mg/dL vs. ≥0.82 mg/dL), baseline hemoglobin (< 11.3 g/dL vs. ≥11.3 g/dL for OS); (< 10.7 g/dL vs. ≥10.7 g/dL for PFS), baseline LDH (< 375 vs. ≥375), baseline serum glucose level (< 110 mg/dL vs. ≥110 mg/dL) and baseline Neutrophil to Lymphocytes Ratio – NLR (<4.6 vs. ≥4.6 for OS; < 5.2 vs. ≥5.2 for PFS). Table S1 shows baseline laboratory values considered in the prognostic analysis. These dichotomized variables were then tested in multivariate analysis, together with the following clinical categorical variables: previous radical surgery, adjuvant treatment, presence of liver metastases, ECOG PS, biliary stent implantation and response to first line therapy. To address the multivariate model overfit and validate the results, a cross‐validation technique, which evaluates the replication stability of the final Cox multivariate model in predicting all outcomes, was also performed, using a resampling procedure. The discriminative ability of the model was assessed by using the Harrell C‐index.

The SPSS (version 21.00), R‐Software (version 3.2.1) statistical programs were used for all analyses.

#### 2.1.1 Prognostic score assessment

The log‐HR acquired from the Cox model was employed to calculate weighting factors for a continuous prognostic index, designed to discern differential risks of outcomes. Coefficient estimates underwent a “normalization” process, dividing by the smallest coefficient and rounding the resulting ratios to the nearest integer value. Consequently, a continuous score was generated, providing an “individualized” risk assessment for each patient.

## 3 RESULTS

### 3.1 Study population and treatment outcomes

From September 2011 to January 2015, 160 metastatic pancreatic cancer patients received AG as second (*n* = 111) or further (*n* = 49) line of treatment. Patients' characteristics are reported in Table 1.

**TABLE 1:** Patients' characteristics in the entire population (*N* = 160) and according the Nab‐Paclitaxel plus Gemcitabine line of treatment.

Characteristics	Overall (*N*=160)	Second line (*N*=111)	Third/fourth line (*N*=49)
AGE (years)			
Median	61	62	60
Range	31–84	31–84	38–79
Sex—*N* (%)			
Male	101 (63.1)	72 (65.9)	29 (59.2)
Female	59 (36.9)	39 (35.1)	20 (40.8)
ECOG PS—d*N* (%)			
0	57 (35.6)	38 (32.2)	19 (38.8)
1	64 (40)	48 (43.2)	16 (32.6)
2	39 (24.4)	25 (22.5)	14 (28.6)
Pancreatic tumor site—*N* (%)			
Head	87 (54.4)	62 (55.9)	25 (51.0)
Body‐tail	73 (45.6)	49 (44.1)	24 (49.0)
Metastatic sites—*N* (%)			
Liver	113 (70.6)	81 (75.7)	32 (60.4)
Lymphnodes	42 (26.2)	32 (29.9)	10 (18.9)
Lung	38 (23.7)	18 (16.8)	20 (37.7)
Peritoneum	33 (20.6)	20 (18.7)	13 (24.5)
Other	15 (9.4)	10 (9.3)	5 (9.4)
Number of metastatic sites—*N* (%)			
1	93 (58.1)	65 (58.6)	28 (57.1)
≥2	67 (41.9)	46 (41.4)	21 (42.9)
Biliary stent—*N* (%)			
Yes	38 (23.7)	25 (22.5)	13 (26.5)
No	122 (76.3)	86 (77.5)	36 (73.5)
Derivative biliary surgery—*N* (%)			
Yes	13 (8.1)	9 (8.1)	4 (8.5)
No	147 (91.9)	102 (91.9)	45 (91.8)
Radical surgery—*N* (%)			
Yes	42 (26.4)	28 (25.2)	14 (28.6)
No	118 (73.8)	83 (74.8)	35 (71.4)
Adjuvant chemotherapy—*N* (%)			
Yes	30 (18.7)	20 (18.0)	10 (20.4)
No	130 (81.3)	91 (82.0)	39 (79.6)
Number of previous lines—*N* (%)			
1	111 (69.4)		
2	42 (26.2)		
>2	7 (4.4)		
First line chemotherapy—*N* (%)			
Gem‐based	64 (40)	35 (31.5)	29 (59.2)
Of which Gem monotherapy	7	5	2
FOLFIRINOX (classic or modified)	96 (60)	76 (68.5)	20 (40.8)

**FIGURE 1:**
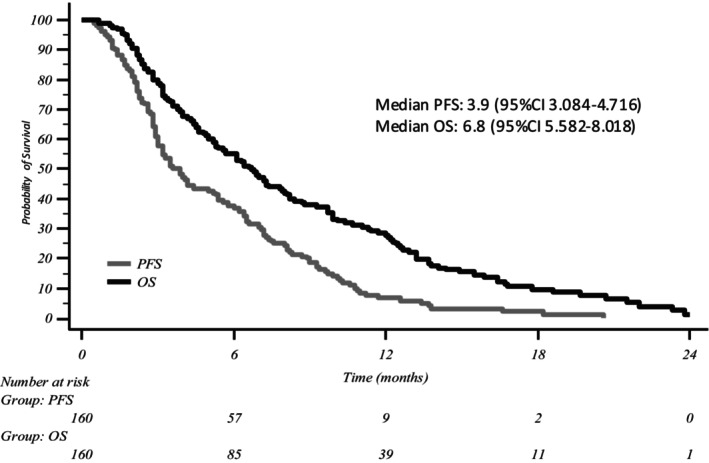
Kaplan–Meier curve for overall survival and progression free survival in the entire population (*N* = 160). OS, overall survival; PFS, progression free survival.

At the time of data censoring, 150 disease progressions and 144 deaths had occurred, and 5 patients were still on treatment (4 in second line), with a median follow up of 7 months (range 1–30). Median OS and median PFS were 6.8 months (95% CI 5.582–8.018) and 3.9 months (95% CI 3.084–4.716); ORR was 22.5% with no CR, DCR was 45.6% with a median duration of DC of 4.7 months; 34.6% of patients experienced a reduction in CA19.9 levels ≥50% (Figure 1; Table 2).

**TABLE 2:** Treatment Outcomes in the entire population (*N* = 160) and according the Nab‐Paclitaxel plus Gemcitabine line of treatment.

Response	Overall (160) *N* (%)	Second line (111) *N* (%)	Third/fourth line (49) *N* (%)
CR	0	0	0
PR	36 (22.5)	25 (22.5)	11 (22.4)
ORR	36 (22.5)	25 (22.5)	11 (22.4)
SD	37 (23.1)	30 (27.0)	7 (14.3)
DCR	73 (45.6)	53 (49.5)	18 (36.7)
PD	87 (54.4)	56 (50.5)	31 (63.3)
Duration of DC (months)			
Median	4.7	5.3	3.95
Range	0.8–15.5	1.0–16.0	2.0–11.0
CA19.9 response Evaluable (*N*=156)			
Increase	85 (54.5)	56 (51.9)	29 (60.4)
Decrease < 50%	17 (10.9)	11 (10.2)	6 (12.5)
Decrease ≥ 50%	54 (34.6)	41 (38.0)	13 (27.1)

Abbreviations: CR, complete response; DC, disease control; DCR, disease control rate; ORR, overall response rate; PD, progression disease; PR, partial response, SD, stable disease.

A separate analysis for patients receiving AG as second or further lines of treatment is shown in Table 2; Table S2, and Figure S1. Focusing the analysis to the 76 patients who received AG as second‐line after FOLFIRINOX, median OS was 5.7 months (95% C.I. 5.0–8.1); median PFS was 3.5 months (95% CI 2.9–4.4) (Figure S2); ORR and DCR were 19.7% and 46.0%, respectively, with a duration of disease control of 5.5 months (Table S3).

### 3.2 Safety

Treatment was well tolerated. Main Grade 3–4 toxicities were neutropenia (25.0%), thrombocytopenia (14.4%), peripheral neuropathy (13.7%) and fatigue (8.1%); their incidence was not significantly different according to AG treatment lines, with the exception of diarrhea that was recorded in 9% of second‐line patients, but not observed in further lines of treatment (*p* = 0.03; Table 3). Dose reduction of one or both drugs was applied in 45% of patients mainly due to hematological toxicity. G‐CSF was used in 25% of patients (primary prophylaxis in 9%), EPO was used in 9.4% of patients. Treatment was permanently discontinued in two patients due to cerebellar ataxia and prolonged neutropenia, respectively. Nab‐Paclitaxel‐related neuropathy showed a trend toward increased incidence in patients treated with a platinum‐based first‐line, but not statistically significant difference was obseved (all‐grade neuropathy: 44% vs. 24%, respectively; *p* 0.14; data not shown).

**TABLE 3:** Treatment‐related Grade 3–4 toxicity in the entire population (*N* = 160) and according the Nab‐Paclitaxel plus Gemcitabine line of treatment.

Toxicity	Overall = 160 G3–G4 N (%)	Second line = 111 G3–G4 N (%)	Third/fourth = 49 G3–G4 *N* (%)	*p* value
ANEMIA—*N* (%)	6 (3.8)	5 (4.5)	1 (2.0)	0.45
Thrombocytopenia—*N* (%)	23 (14.4)	15 (13.5)	8 (16.3)	0.64
Neutropenia—*N* (%)	40 (25.0)	30 (27.0)	10 (20.4)	0.37
Fatigue—*N* (%)	13 (8.1)	10 (9.0)	3 (6.1)	0.54
Mucositis—*N* (%)	5 (3.1)	3 (2.7)	2 (4.1)	0.64
Neurotoxicity—*N* (%)	23 (13.7)	19 (17.8)	4 (7.6)	0.08
Nausea/vomiting—*N* (%)	6 (3.8)	4 (3.6)	2 (4.1)	0.88
Diarrhea—*N* (%)	10 (6.3)	10 (9.0)	0	0.03

### 3.3 Prognostic analysis

The prognostic impact of pre‐treatment clinical and laboratory parameters on survival endpoints was explored in the entire population. Among clinical/laboratory factors tested, LDH, fasting serum glucose, NLR, PS, liver metastases, and PFS to first‐line therapy had an independent impact on OS at multivariate analysis (Table 4); similarly, LDH, fasting serum glucose, NLR, and PFS to first‐line therapy were also significantly associated with PFS (Table 4).

**TABLE 4:** Multivariate analysis for overall survival and progression free survival.

	OS			PFS		
Variable	HR	95% CI	*p* value	HR	95% CI	*p* value
Liver metastases (yes vs. no)	1.704	1.078–2.692	0.022	–	–	–
ECOG PS (2 vs. 0–1)	1.769	1.081–2.895	0.023	–	–	–
LDH (≥375 vs. < 375)	2.855	1.684–4.840	< 0.0001	2.716	1.636–4.510	<0.0001
NLR (≥4.6 vs. <4.6 for OS) (≥5.2 vs. <5.2 for PFS)	1.553	0.879–2.743	0.130	2.419	1.410–4.152	0.001
Fasting serum glucose (≥110 vs. < 110)	1.766	1.100–2.837	0.019	1.619	1.010–2.597	0.046
1st Line PFS (<11 month vs. ≥11 month)	4.940	2.337–10.442	< 0.0001	2.419	1.339–4.373	0.003

Abbreviations: OS, overall survival; PFS, progression free survival.

**FIGURE 2:**
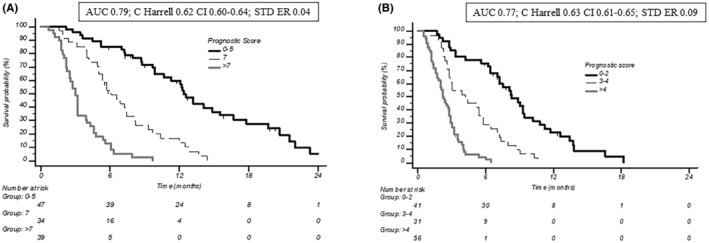
(A) Overall survival and (B) progression free survival according to the prognostic model. AUC, area under curve.

Individual factors entering the final OS/PFS prognostic models were validated by bootstrap resampling analysis, with a replication rate ≥ 75% for all factors, with the exception of NLR for OS **(**Table S4). In order to build prognostic nomograms for OS and PFS, individual scores were attributed to each variable, based on their weighted impact in the multivariate model (Table S5). A three‐class model (score: ≤5, 6/7, >7) identifying patients at significantly different probability of survival was derived (Figure 2A; Table S6); a similar three‐class model (scores: ≤2, 3/4, >4) effectively identified patients at different probability of being free from progression (Figure 2B; Table S7). These differences were statistically significant (*p* for both risk models: < 0.0001). When the three‐class model was applied to patients treated with AG in second line after FOLFIRINOX (*n* = 76), the prognostic value of the identified variables was confirmed (Figure S3).

## 4 DISCUSSION

In this retrospective study, clinical outcomes of metastatic pancreatic cancer patients who received AG as second or further line of treatment were evaluated. In line with other published results,^29^, ^30^, ^31^, ^32^, ^33^, ^34^ AG was well tolerated in a cohort of pre‐treated patients, showed clinical activity with an ORR of 22.5%, a DCR of 45.6%, and resulted in median PFS and OS of 3.9 and 6.8 months, respectively. In particular, in the subgroup of patients who received AG as a second‐line therapy (*n* = 111, 66.9%), median PFS and OS were 4.2 and 7.4 months, respectively. Furthermore, in the 76 (68%) patients treated with AG as second‐line treatment after first‐line FOLFIRINOX, a 19.7% ORR and 46.0% of DCR were observed, resulting in a median PFS and OS of 3.5 and 5.7 months, respectively.

Second‐line treatment in mPC remains an unmet medical need. Available phase III randomized trials who compared the OFF, FOLFOX‐6 and nal‐IRI/FUFA combinations versus fluoropyrimidine monotherapy have been conducted in patients who had been pre‐treated mostly with gemcitabine monotherapy (100%, 77.8%, and 44.6%, respectively) or gemcitabine‐based combinations (0%, 22.2, and 55.4%, respectively), not including AG.^10^, ^19^, ^20^, ^21^ Differently, only few retrospective data are available about potential activity of AG in gemcitabine pretreated patients.^36^ In the current mPC treatment landscape, in which AG represents the preferred and widely used first‐line regimen, patients progressed after this regimen, may benefit of fluoropyrimidines‐based second‐line treatment as confirmed by a post‐hoc analysis of the randomized, phase III, MPACT trial as well as in restrospective series.^17^, ^18^ In particular, single agent fluoropyrimidines, doublets with oxaliplatin, irinotecan, nanoliposomal irinotecan and in few, selected patients, triplets (i.e., FOLFIRINOX, classic or modified) have shown some efficacy in recent real‐life experiences.^37^, ^38^ Differently, in the patients progressed after FOLFIRINOX first‐line, at present, there is no randomized trial comparing different second‐line strategies: in the PRODIGE 4 study, 47% of patients received second‐line treatment, mostly gemcitabine (82.5%) or gemcitabine‐based combinations (12.5%), not including AG.^4^ Retrospective studies reported some activity for single‐agent gemcitabine,^25^, ^26^, ^27^, ^28^ suggesting that some patients (i.e., younger patients with good performance status) might benefit more than the others.^25^, ^26^, ^28^ Single agent *nab*‐Paclitaxel has been evaluated in a phase II study by Hosein et al.,^39^ not including patients treated with first line FOLFIRINOX, and small retrospective series^40^, ^41^ which included a small number of FOLFIRINOX‐pretreated patients, showing activity, even in heavily pretreated patients. AG has been tested in two, single arm, phase II studies in Asian patients progressing from FOLFIRINOX, demonstrating an ORR of 13%–15%, a median PFS of 3.8–5.8 months, and a median OS of 7.6–9.9.^29^, ^30^ Small retrospective series also evaluated AG in patients progressing from first line FOLFIRINOX with median PFS in the range of 3.8–5.6 months and median OS in the range of 8.8–15.6 months.^31^, ^32^, ^33^ Our retrospective data with the AG regimen as second‐line treatment are in line with previous experiences in both the gemcitabine‐ and FOLFIRINOX‐pretreated populations; however, activity in gemcitabine‐pretreated patients currently bears little interest as the vast majority of mPC patients receives AG as their first‐line treatment.^7^ A small, retrospective, Canadian series suggested a potential advantage for AG as compared to gemcitabine monotherapy in the post‐FOLFIRINOX setting^42^; this trend was recently confirmed in a large (*n* = 427), retrospective analysis, comparing AG (*n* = 219) versus gemcitabine alone (*n* = 208) in metastatic pancreatic cancer patients progressing from first‐line FOLFIRINOX.^34^ This study showed a significant advantage for AG in both PFS and OS (HR for PFS: 0.53, 95% CI: 0.43–0.65; *p* < 0.0001); (HR for OS: 0.67; 95% CI: 0.53–0.86; *p* < 0.0001), across all the prespecified subgroups; improved disease control rate (55.9% vs. 32.5%; *p* < 0.001) and ORR (11.3% vs. 8.3%) were also observed with AG as compared to gemcitabine alone. In multivariate analysis, AG, PS and longer first‐line time‐to‐progression were independent predictors of survival outcomes (PFS and OS). Currently, a phase III randomized study is recruiting patients to evaluate AG versus gemcitabine alone as second‐line treatment after first line FOLFIRINOX (NCT03943667).

Regardless of the specific regimen employed, the outcomes of second‐line chemotherapy remain generally poor in metastatic pancreatic cancer and its benefit in individual patients is highly variable. Therefore, a careful evaluation and fair discussion of potential risks and benefits of second‐line treatment with patients and their families is mandatory and, perhaps, more important than the specific regimen selected. Individual prognostic factors have been proposed in this setting (PS, tumor burden, treatment response and duration of first‐line therapy, second‐line therapy regimen, and circulating biomarkers) and attempts have been made to integrate them into prognostic nomograms.^43^, ^44^ In a large retrospective Korean series, a nomogram for predicting OS on second‐line chemotherapy for mPC was devised based on number of metastatic lesions, presence of peritoneal metastases, occurrence of thrombotic events during first line, and CA19.9 levels; Harrel's C values for the development and validation cohorts were 0.62 and 0.56, respectively.^43^ In this series patients had received gemcitabine‐based adjuvant or first line treatment (which were considered together) and went on to receive fluoropyrimidine‐based (FOLFORINOX, FOLFOX, Cape/OX, or folinic acid/etoposide/cisplatin—FEP) second line. In another series from Taiwan,^43^ eight variables (sex, ECOG performance status, reasons for first line discontinuation, first‐line duration, NLR, tumor status [locally advanced versus metastatic], BMI, and serum CA19‐9 levels) entered the final second‐line prognostic model; Harrel's C values for the development and validation cohorts were 0.73 and 0.72, respectively. In this series, locally advanced (approximately 30%) and metastatic patients had received gemcitabine‐based first line treatment and went on to receive either fluoropyrimidine‐ or gemcitabine‐based second line. Our series included a relatively more homogeneous population of metastatic pancreatic cancer patients, treated with first‐line FOLFIRINOX in 60% and 68% of the cases in the overall and second‐line populations, respectively, all receiving AG as their second or further treatment line; similar to previously published experiences, four core factors (LDH and fasting serum glucose levels, NLR, and first‐line PFS) entered both PFS and OS prognostic models, while ECOG PS and the presence of liver metastases additionally impacted on OS prediction. Overall the three‐risk classes model devised had an excellent accuracy (AUC: 0.77 and 0.79; Harrels C: 0.62 and 0.63, for PFS and OS, respectively) in predicting survival outcomes upon second‐ or further‐line AG and retained its predictive value in the more homogeneous, second‐line, FOLFIRINOX‐pretreated population. These data are consistent with a recent retrospective study conducted in 103 patients receiving AG as second‐line therapy after FOLFIRINOX, which identified two different prognostic categories (good and poor) according to ECOG PS, NLR, and modified Glasgow Prognostic Score (mGPS).^45^

## 5 CONCLUSIONS

Despite of the limitations of a retrospective cohort study, the data presented here support the use of second‐ or further‐line AG as a reasonable treatment option for metastatic pancreatic cancer patients. Particularly, in the context of the current first‐line treatment landscape, AG could be considered for patients who have progressed after receiving first‐line FOLFIRINOX. Notably, in this subgroup, we have developed a prognostic model that allows the differentiation of patients into distinct risk categories. Patients with an excellent prognosis (good risk) have shown a median progression‐free survival (PFS) of 8.3 months and a median overall survival (OS) of 12.4 months, making AG treatment a valuable choice. Conversely, patients with a poor prognosis (poor risk) exhibited a median PFS of 2.2 months and a median OS of 3 months, indicating that alternative treatment options, such as best supportive care, may be more appropriate. This prognostic model, once validated prospectively, could serve as a practical tool for guiding daily clinical decision‐making and providing valuable information for discussions with pretreated metastatic pancreatic cancer patients and their families.

## AUTHOR CONTRIBUTIONS

**Guido Giordano:** Conceptualization (lead); data curation (equal); formal analysis (equal); funding acquisition (lead); investigation (equal); methodology (equal); project administration (equal); resources (equal); software (supporting); supervision (equal); validation (equal); visualization (equal); writing – original draft (lead); writing – review and editing (equal). **Michele Milella:** Conceptualization (equal); data curation (equal); formal analysis (equal); funding acquisition (equal); investigation (equal); methodology (equal); project administration (equal); resources (equal); software (supporting); supervision (equal); validation (equal); visualization (equal); writing – original draft (equal); writing – review and editing (equal). **Matteo Landriscina:** Data curation (equal); investigation (equal); resources (equal); writing – original draft (equal); writing – review and editing (supporting). **Francesca Bergamo:** Data curation (equal); investigation (equal); resources (equal); visualization (equal). **Giuseppe Tirino:** Data curation (equal); investigation (equal); resources (equal); visualization (equal). **Antonio Santaniello:** Data curation (equal); investigation (equal); resources (equal); visualization (equal). **Alberto Zaniboni:** Data curation (equal); investigation (equal); resources (equal); visualization (equal). **Enrico Vasile:** Data curation (equal); investigation (equal); resources (equal); visualization (equal). **Ferdinando De Vita:** Data curation (equal); investigation (equal); resources (equal); visualization (equal). **Giovanni Lo Re:** Data curation (equal); investigation (equal); resources (equal); visualization (equal). **Vanja Vaccaro:** Data curation (equal); investigation (equal); resources (equal); visualization (equal). **Elisa Giommoni:** Data curation (equal); investigation (equal); resources (equal); visualization (equal). **Donato Natale:** Data curation (equal); investigation (equal); resources (equal); visualization (equal). **Raffaele Conca:** Data curation (equal); investigation (equal); resources (equal); visualization (equal). **Daniele Santini:** Data curation (equal); investigation (equal); resources (equal); visualization (equal). **Luigi Maiorino:** Data curation (equal); investigation (equal); resources (equal); validation (equal). **Gianni Sanna:** Data curation (equal); investigation (equal); resources (equal); visualization (equal). **Vincenzo Ricci:** Data curation (equal); investigation (equal); resources (equal); visualization (equal). **Aldo Iop:** Data curation (equal); investigation (equal); resources (equal); visualization (equal). **Vincenzo Montesarchio:** Data curation (equal); investigation (equal); resources (equal); visualization (equal). **Letizia Procaccio:** Data curation (equal); investigation (equal); resources (equal); visualization (equal). **Silvia Noventa:** Data curation (equal); investigation (equal); resources (equal); visualization (equal). **Roberto Bianco:** Data curation (equal); investigation (equal); resources (equal); visualization (equal). **Antonio Febbraro:** Data curation (equal); investigation (equal); resources (equal); visualization (equal). **Sara Lonardi:** Data curation (equal); investigation (equal); resources (equal); visualization (equal). **Giampaolo Tortora:** Data curation (equal); investigation (equal); resources (equal); validation (equal). **Isabella Sperduti:** Conceptualization (equal); data curation (equal); formal analysis (lead); investigation (equal); methodology (equal); project administration (equal); resources (equal); software (lead); supervision (equal); validation (equal); visualization (equal); writing – original draft (equal); writing – review and editing (equal). **Davide Melisi:** Conceptualization (equal); data curation (equal); formal analysis (equal); funding acquisition (equal); investigation (equal); methodology (equal); project administration (equal); resources (equal); software (supporting); supervision (lead); validation (equal); visualization (equal); writing – original draft (equal); writing – review and editing (lead).

## FUNDING INFORMATION

The present study was supported by the “5 per mille” 2018–2019 LILT Investigator Grant; the Italian Ministry of University and Research under PNRR‐M4C2‐ I1.3 Project PE_00000019 “HEAL ITALIA.”

## CONFLICT OF INTEREST STATEMENT

Guido Giordano reports: travel and accommodation expenses by Celgene and Astra Zeneca, Honoraria for participation to Advisory Boards from Celgene, Servier, Astra Zeneca and MSD. Michele Milella reports: research funding (to institution) from Roche; travel and accommodation expenses by Astra Zeneca; consulting fees from Astra Zeneca and MSD; Honoraria for participation to Advisory Boards from Astra Zeneca, MDS, Ipsen, Sevier and Viatris. Letizia Procaccio reports: personal fee for scientific consultancy from Astra Zeneca. Sara Lonardi reports: research funding (to Institution) from Amgen, Astellas, Astra Zeneca, Bayer, Bristol‐Myers Squibb, Daichii Sankyo, Hutchinson, Incyte, Merck Serono, Mirati, MSD, Pfizer, Roche, Servier; personal honoraria as invited speaker from Amgen, Bristol‐Myers Squibb, Incyte, GSK, Lilly, Merck Serono, MSD, Pierre‐Fabre, Roche, Servier; participation in advisory board for Amgen, Astellas, Astra Zeneca, Bayer, Bristol‐Myers Squibb, Daiichi‐Sankyo, GSK, Incyte, Lilly, Merck Serono, MSD, Servier, Takeda. Davide Melisi reports: Research Grants from: Celgene, Evotec, Incyte, iOnctura, Roche, Servier, Shire; Consulting fees from: Eli Lilly, Evotec, Incyte, iOnctura, IQVIA, Servier, Shire, Taiho; Honoraria for participation to Advisory Boards from: Incyte, Roche, Servier, Taiho, Terumo; Payment for lectures or presentations from: Baxter, Incyte, MSD Italia, Roche.

## INFORMED CONSENT STATEMENT

All participants gave their written informed consent in accordance with Declaration of Helsinki.

## IRB STATEMENT

The Coordinating Sites Institutional board (CE Lazio 1, Prot. 1439/2014) approved the study.

## Supporting information


**Data S1:** Supporting information.

## Data Availability

All data generated or analyzed during this study are included in this published article [and its supplementary information files] and are available upon reasonable request.
