# Supplementary material for: Prognostic analysis and outcomes of metastatic pancreatic cancer patients receiving nab‐paclitaxel plus gemcitabine as second or later‐line treatment
Source: Cancer Med. 2024 Jun 25;13(12):e7345. doi: 10.1002/cam4.7345 (PMC11199338; doi:10.1002/cam4.7345)
Supplement: Supplementary file 1 — Data S1: Supporting information. [file CAM4-13-e7345-s001.docx]

| **BASELINE VALUE** | **OVERALL**  **(N=160)** | **SECOND LINE**  **(N=111)** | **3rd/4th LINE**  **(N=49)** |
| --- | --- | --- | --- |
| **HAEMOGLOBIN**  ***Median***  ***Range*** | 11.4 g/dl  8.5 – 16.3 g/dl | 11.6 g/dl  8.5 – 16.3 g/dl | 11.1 g/dl  9.1 – 16 g/dl |
| **BIRILUBIN**  ***Median***  ***Range*** | 0.77 mg/dl  0.20 – 4.78 mg/dl | 0.70 mg/dl  0.20 – 4.78 mg/dl | 0.94 mg/dl  0.30 – 2.16 mg/dl |
| **LDH**  ***Median***  ***Range*** | 347 U/I  115 – 1657 U/I | 320 U/I  133 – 1657 U/I | 415 U/I  115 – 827 U/I |
| **NLR**  ***Median***  ***Range*** | 3.5  0.6 – 12.3 | 3.0  0.6 – 12.3 | 4.6  0.9 – 10.6 |
| **CA 19.9**  ***Median***  ***Range*** | 2570 U/I  1.1 – 698000 U/I | 2650 U/I  1.1 – 698000 U/I | 3045 U/I  6.6 – 448000 U/I |

**Supplementary Table S1. Baseline laboratory values considered in the prognostic analysis.**

**NLR: Neutrophil to Lymphocytes Ratio**

| **OUTCOME** | **OVERALL**  **N=160** | **2^nd^ LINE**  **N=111** | **3^rd^/4^th^ LINE**  **N=49** |
| --- | --- | --- | --- |
| **Median OS months**  **(95% C.I.)** | 6.8  (5.582-8.018) | 7.4  (5.937-8.863) | 4.9  (1.856-7.944) |
| ***6 months OS rate (%)*** | 53.1% | 56.8% | 44.9% |
| ***12 months OS rate (%)*** | 25.0% | 29.7% | 14.3% |
| ***18 months OS rate (%)*** | 6.9% | 8.1% | 4.1% |
| **Median PFS months**  **(95% C.I.)** | 3.9  (3.084-4.716) | 4.2  (2.501-5.899) | 2.9  (2.140-3.660) |
| ***6 months PFS rate (%)*** | 35.6% | 40.5% | 26.5% |
| ***12 months PFS rate (%)*** | 5.6% | 5.4% | 6.1% |

**Supplementary Table S2. Survival Outcomes in the entire population (N=160) and according the Nab-Paclitaxel plus Gemcitabine line of treatment.**

*Probability of Survival*

**Supplementary Figure S1A, 1B. Overall Survival and Progression Free Survival according to the line of treatment.**

*Probability of Survival*

**Supplementary Figure S2. Overall Survival and Progression Free Survival curves in the post-FOLFIRINOX second-line population (n=76).**

| **RESPONSE** | **AG 2^nd^ line after FOLFIRINOX (76)**  **N (%)** |
| --- | --- |
| **CR** | 0 |
| **PR** | 15 (19.7) |
| **ORR** | 15 (19.7) |
| **SD** | 20 (26.3) |
| **DCR** | 35 (46.0) |
| **PD** | 41 (54.0) |
| **DURATION OF DC (months)**  **Median**  **Range** | 5.5  1.0 – 14.0 |
| **CA19.9 response**  **Evaluable (N=73)**  **Increase**  **Decrease < 50%**  **Decrease ≥ 50%** | 36 (49.3)  7 (9.6)  30 (41.1) |

**Supplementary Table S3. Nab-Paclitaxel and Gemcitabine activity in post- FOLFIRINOX second-line population. CR, Complete Response; PR, Partial Response, ORR, Overall Response Rate; SD, Stable Disease; DCR, Disease Control Rate; PD, Progression Disease; DC, Disease Control**

| **VARIABLE** | **%REPLICATION RATE (PFS)** | **%REPLICATION RATE (OS)** |
| --- | --- | --- |
| LIVER MTS | - | 80 |
| PS | - | 80 |
| 1 line PFS (11 months) | 100 | 100 |
| LDH (375) | 100 | 100 |
| Glucose (110) | 75 | 90 |
| NLR (5.2 /4.6) | 85 | 65 |

**Supplementary Table S4. Bootstrap validation of individual prognostic factors. PFS, Progression Free Survival; OS, Overall Survival; MTS, metastases; PS, Performance Status; NLR, Neutrophil to Lymphocite Ratio**

|  | **Score Points** | | | |
| --- | --- | --- | --- | --- |
|  | **0** | **1** | **2** | **4** |
| **PFS** | | | | |
| LDH | ≤375 U/I |  | >375 U/I |  |
| Fasting Serum Glucose | ≤110 mg/dl | >110 mg/dl |  |  |
| NLR | ≤5.2 |  | >5.2 |  |
| 1^st^ line PFS | ≥11 months |  | ≤11 months |  |
|  |  |  |  |  |
| **OS** | | | | |
| LDH | ≤375 U/I |  | >375 U/I |  |
| Fasting Serum Glucose | ≤110 mg/dl | >110 mg/dl |  |  |
| NLR | ≤ 4.6 | >4.6 |  |  |
| ECOG PS | 0-1 | 2 |  |  |
| Liver mts | No | Yes |  |  |
| 1^st^ line PFS | ≥11 months |  |  | <11 months |

**Supplementary Table S5. Weighted prognostic scoring according to outcome.**

|  | **6-mo OS**  **%** | **12-mo OS**  **%** | **18-mo OS**  **%** | **Median OS** | **P value** |
| --- | --- | --- | --- | --- | --- |
| **0-5 (47 pts)** | **85.1** | **49.7** | **27.3** | **12.4 (11.3-13.5)** | **<0.0001** |
| **6-7 (34 pts)** | **49.3** | **13.2** | **0** | **6.1 (4.4-7.8)** |  |
| **>7 (39 pts)** | **5.1** | **0** | **0** | **3.0 (2.6-3.4)** |  |

**Supplementary Table S6. Overall Survival (median and rates according to prognostic score). OS, Overall Survival; mo, months; pts, patients.**

|  | **6-mo PFS**  **%** | **Median PFS** | **P value** |
| --- | --- | --- | --- |
| **0-2 (41 pts)** | **68.1** | **8.3 (6.6-10.0)** | **<0.0001** |
| **3-4 (31 pts)** | **29.0** | **4.2 (2.1-6.3)** |  |
| **>4 (56 pts)** | **1.9** | **2.2 (1.7-2.7)** |  |

**Supplementary Table S7. PFS (median and rates according to prognostic score). PFS, Progression Free Survival; mo, months; pts, patients.**

*Probability of Survival*

**A**

*P < 0.0001*

**Supplementary Figure S3. Overall Survival (Fig. S3A) and Progression Free Survival (Fig. S3B) according to the prognostic model in the post- FOLFIRINOX second-line AG population.**
